# Primary squamous cell carcinoma of thyroid gland: 11 case reports and a population-based study

**DOI:** 10.1186/s12957-022-02814-9

**Published:** 2022-11-03

**Authors:** Wanyun Yan, Huiying Chen, Jiangmiao Li, Ruifa Zhou, Jiping Su

**Affiliations:** 1 Department of Otolaryngology-Head and Neck Surgery, The First Affiliated Hospital of Guangxi Medical University, Nanning, 530021 Guangxi China; 2 Present Address: Department of Otolaryngology-Head and Neck Surgery, Nanxishan Hospital of Guangxi Zhuang Autonomous Region, Guangxi Guilin, China

**Keywords:** Primary thyroid tumor, Squamous cell carcinoma, Prognosis, Case report, Clinical features

## Abstract

**Background:**

Primary squamous cell carcinoma of thyroid gland (PSCCT) is a highly aggressive malignant tumor associated with a poor prognosis. Due to the rare case, there is a knowledge gap on the features of PSCCT. There is limited understanding of the treatment and molecular biology of this tumor. More genomic work and relevant perspective work need to be done.

**Methods:**

We retrospectively reviewed the medical information of patients with PSCCT diagnosed from December 2009 to December 2020 at The First Affiliated Hospital of Guangxi Medical University. In addition, we conducted an electronic search of the paper in CNKI, Wanfang, VIP, PubMed, Embase, Web of Science, and ProQuest databases by recently updated articles. Survival analysis was conducted using the Kaplan–Meier method.

**Results:**

There were only 11 patients met the study’s inclusion criteria in our institution. The patients ranged in age from 25 to 68 years old and female preponderance (*M:F* = 1:1.7). The median survival time was 6 months, and 1-year survival rate was 33.3%. Fifty-three patients’ individual data from 45 articles were selected for analysis. The median age at diagnosis was 63 years and female preponderance (*M:F* = 1:2.5). The commonest complaint was the anterior neck mass (77.3%), followed by hoarseness (32.1%). The median survival time was 9 months, and the overall 1-, 2-, and 5-year survival rate was 39.8%, 33.7%, and 26.9%, respectively. The log-rank method shows that age, tumor size, lymph node status, M stage, surgical range, and tracheal status were the relevant factors affecting the prognosis. In contrast, gender, treatment modality, and resection margin were not prognostic factors. On multivariable analysis, age and M stage were associated with overall survival.

**Conclusion:**

The median overall survival was 6–9 months of PSCCT. Age and M stage are predictors of PSSCT.

**Supplementary Information:**

The online version contains supplementary material available at 10.1186/s12957-022-02814-9.

## Background

PSCCT is a rare type of thyroid cancer that accounts for 0.1–0.4% of all cases [1, 2]. It is a highly aggressive malignant tumor associated with a poor prognosis. The overall 1-year survival rate was 32.2%, the 3-year survival rate was 16.5%, and 5-year survival rate was 15%. The median survival time was 7.7–9.1 months [2, 3]. Patients with PSCCT often present at an advanced stage due to high rates of regional metastasis (55.4%), extrathyroidal extension (64%), and distant metastasis (11.7%) [2]. The female-to-male ratio is 1.37–2.4 [1, 4]. The mean age at diagnosis for PSCCT patients was 68 years [2, 3]. To address this, we retrospectively identified patients with PSCCT who received treatment at The First Affiliated Hospital of Guangxi Medical University during 2009–2020 and reviewed published PSCCT literature, to understand the clinical characteristics and identify the prognostic factors of PSCCT.

## Methods

### Inclusion criteria

- Rough/or fine-needle aspiration biopsy or postoperative histopathology to confirm a diagnosis of PSCCT
- Complete clinical data, including gender, age, chief complaint, pathology, the tumor size, lymph node status, M stage, trachea status, surgical range, resection margin, treatment modality, and prognosis.

### Exclusion criteria

- Metastatic/secondary squamous cell carcinoma histopathology
- Incomplete clinical data. In addition, patients with other malignant tumors at the same time were excluded from published PSCCT cases.

### Patient demographics

The ethics board of The First Affiliated Hospital of Guangxi Medical University approved the protocol the study on March 28, 2022 (no. 2022-KY-E-086). All patients or their dependents provided written informed consent for participation in the study. With approval from our institutional review board, we conducted a retrospective review of medical records in our institute. From December 2009 to December 2020, only 11 patients met the study’s inclusion criteria. All patients were staged according to the American Joint Committee on Cancer 8 (2018) based on pathologic characteristics.

### Search strategy

**Fig. 1:**
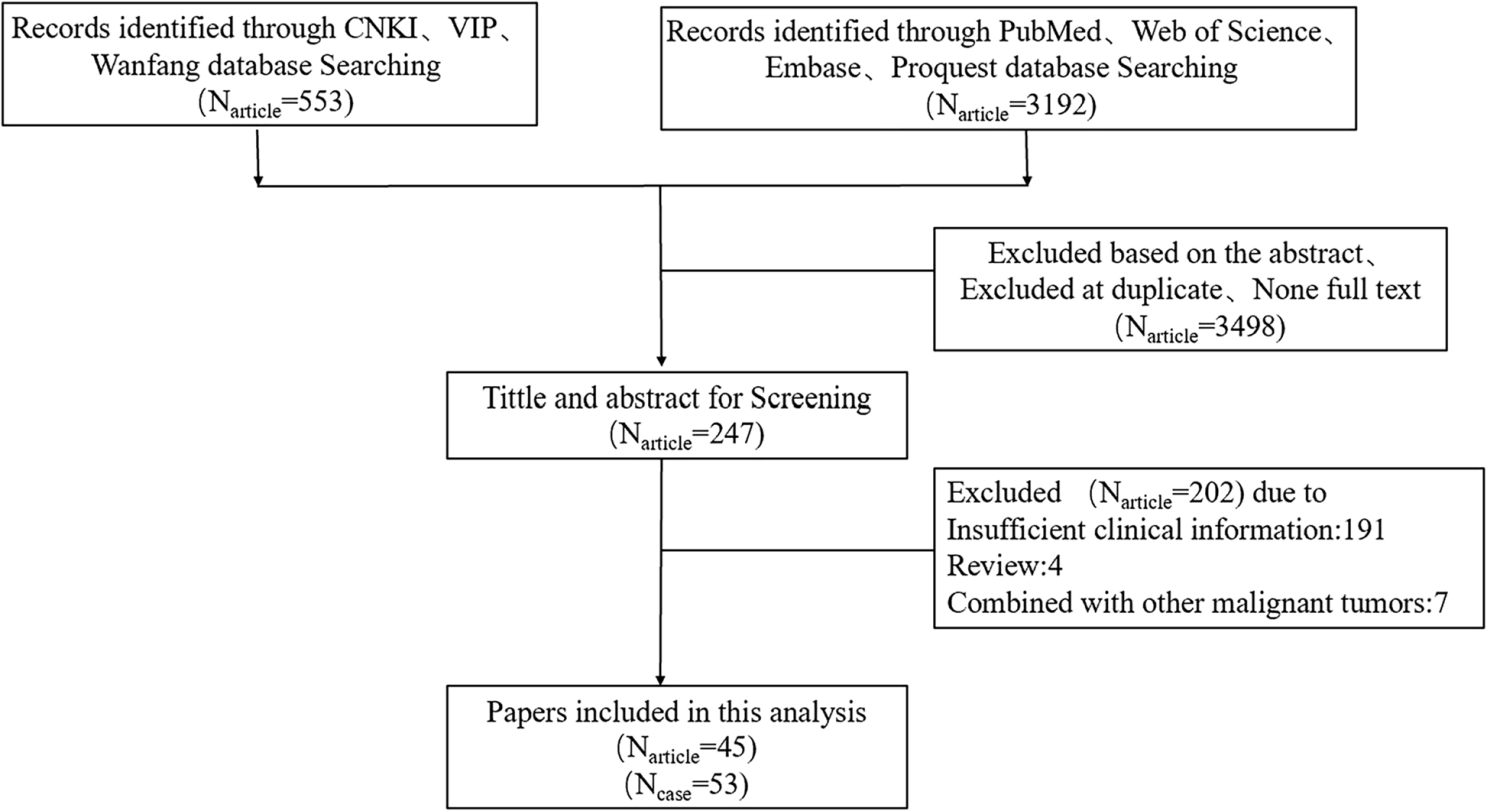
Literature search and study diagram

A systematic search was conducted of CNKI, Wanfang, VIP, PubMed, Embase, Web of Science, and ProQuest databases (from inception to December 2021) for publications using the keywords “primary,” “squamous,” “carcinoma,” “cancer,” “neoplasm,” and “thyroid”. After review, 45 papers (see Additional file 1) were selected by the inclusion/exclusion criteria, and these papers contributed information for 53 patients with PSCCT (Fig. 1).

### Tumor case cohort

The data collected in this study covered decades; we considered tumor size as a variable and analyzed its significance for prognosis. The lymph node status criteria of surgical patients and nonsurgical patients are different. Surgical patients were grouped according to the pathological results and surgical records. N1 group: lymph nodes had metastasis diagnosed by pathology after the operation. N0 group: (1) lymph node without metastasis was diagnosed by pathology after operation; (2) the literature recorded no lymph node metastasis during the operation. The nonsurgical patient group was divided according to the imaging results. N0 group: no lymph node metastasis was diagnosed by imaging. N1 group: lymph node metastasis was diagnosed by imaging. The M-stage criteria of all patients are the clinical stage.

Given that we did not have a large amount of data in the selected literature, we converted the resection margin into dichotomous variables. R1 group is as follows: (1) the tumor extended to the tissue margins diagnosis by pathology after operation, (2) macroscopic residual tumor recorded in literature, (3) patients for nonsurgical treatment, and (4) the primary tumor has distant metastasis. Considering that the above patients have a residual tumor, we uniform these patients into R1 group. Conversely, patients without residual tumors recorded in literature are divided into the R0 group.

### Statistical analysis

Survival analysis was performed using Kaplan–Meier estimates. A univariable Cox proportional hazard regression model was used to identify independent predictors of OS in patients with PSCCT, and those found to be statistically significant were subsequently used in a multivariable analysis. A *P*-value of < 0.05 was considered statistically significant. All statistical analyses were performed using SPSS software version 26.0 (IBM Corp.). The variables were considered age, gender, tumor size, lymph node status, M stage, tracheal status, resection margin, treatment modality, and surgical range.

## Results

### Clinical characteristics

In our institution, 11 eligible patients were of Asian ethnicity. The median age at diagnosis was 44 years (range from 25 to 68) and 4 males and 7 females (Table 1). The 7th patient underwent surgery for thyroid papillary carcinoma 2 years ago, and cervical lymph node metastasis was found in operation. However, due to personal reasons, no further treatment was performed after the operation. The 11th patient was diagnosed with nasopharyngeal carcinoma 10 years ago and received chemoradiotherapy. During this period, complications such as radiation pneumonitis, hypothyroidism, and hearing loss occurred. The past medical history of other patients is normal. The mean size of the primary tumor was 47.6 mm. A total of 18.2% of the patients in this study were older than 60 years. The common clinical symptoms were neck mass (9/11), neck pain (5/11), and hoarseness (4/11). In our study, 27.3% (3/11) of the PSCCT cases were in the left lobe, 45.4% (5/11) were in the right lobe, and 27.3% (3/11) were bilateral.

**Table 1:** Clinicopathological features and survival

No.	Age	Gender status	T size	pTNM	Treatment	Survival	Current
1	63	F	4.1	T4bN1M0	S/C/R	*	*
2	39	M	5.2	T4aN1M0	S	*	*
3	25	F	3.5	T2N0M0	S	48	NED
4	54	M	6.6	T4bNxM0	S	3	DOD
5	68	F	5.3	T4bN1M0	S	13	DOD
6	40	F	2.2	T4aN1M0	S/C	7	NED
7	44	F	8.8	T4aNxM0	S	6	DOD
8	52	F	3.0	T4aN1M0	S/I	10	DOD
9	43	M	6.0	T4aN1M0	S	*	*
10	60	M	4.5	T3aNxM0	N	3	DOD
11	44	F	3.2	T4aNxM0	N	0.1	DOD

*C* Chemotherapy, *R* Radiotherapy, *S* Surgery, *I* Iodine radiation therapy, *NED* No evidence of disease, *DOD* Died of disease, *lost follow-up, *N* refuse treatment

According to selected papers, the median age at diagnosis for 53 eligible patients was 63 years (range from 24 to 88), with 15 males and 38 females (*M:F* = 1:2.5). A total of 58.5% of patients in the study were older than 60 years. The mean size of the primary tumor was 49.9 mm. Patients with the carcinoma typically present with neck mass (77.3%), hoarse voice (32.1%), dysphagia (20.7%), neck pain (16.9%), and dyspnea (16.9%). Interestingly, 37.7% (20/53) patients had papillary thyroid carcinoma, and 7.5% (4/53) had follicular thyroid carcinoma synchronously. The present analysis showed that 67.9% (36/53) of reported patients had lymph node metastases confirmed by pathology, 28.3% (15/53) presented with tracheal invasion or oppression, and 7.5% (4/53) presented with lung metastasis.

### Ultrasonographic characteristics

**Fig. 2:**
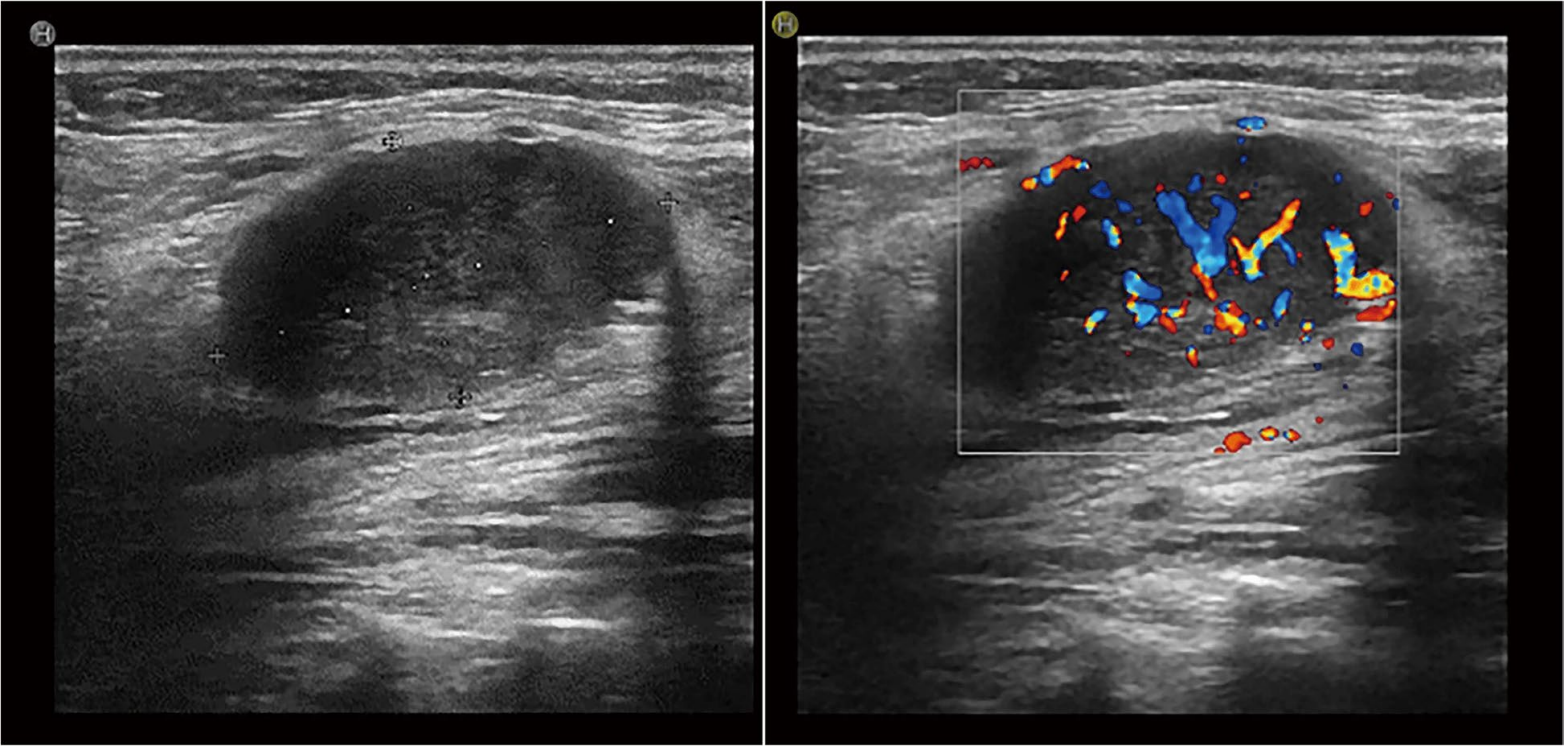
Ultrasound showed that a 2.2 cm × 1.7 cm nodule with blood flow signals

There were 7 patients who underwent B-ultrasound examination in our institution, and 71.4% (5/7) of patients presented with a malignant tumor. The average size of PSCCT was 41.7 mm. Ultrasound showed that 71.4% (5/7) of the thyroid was enlarged with morphologically abnormal, and 57.1% (4/7) had calcification. Approximately, 85.7% (6/7) of the PSCCT was hypoechoic or mixed, presenting uneven echo. Color Doppler flow imaging demonstrated blood flow signals in all nodules (Fig. 2). There were 4 patients who had cervical lymph node metastasis diagnosed by ultrasound and confirmed by pathology in the end.

### Imaging

**Fig. 3:**
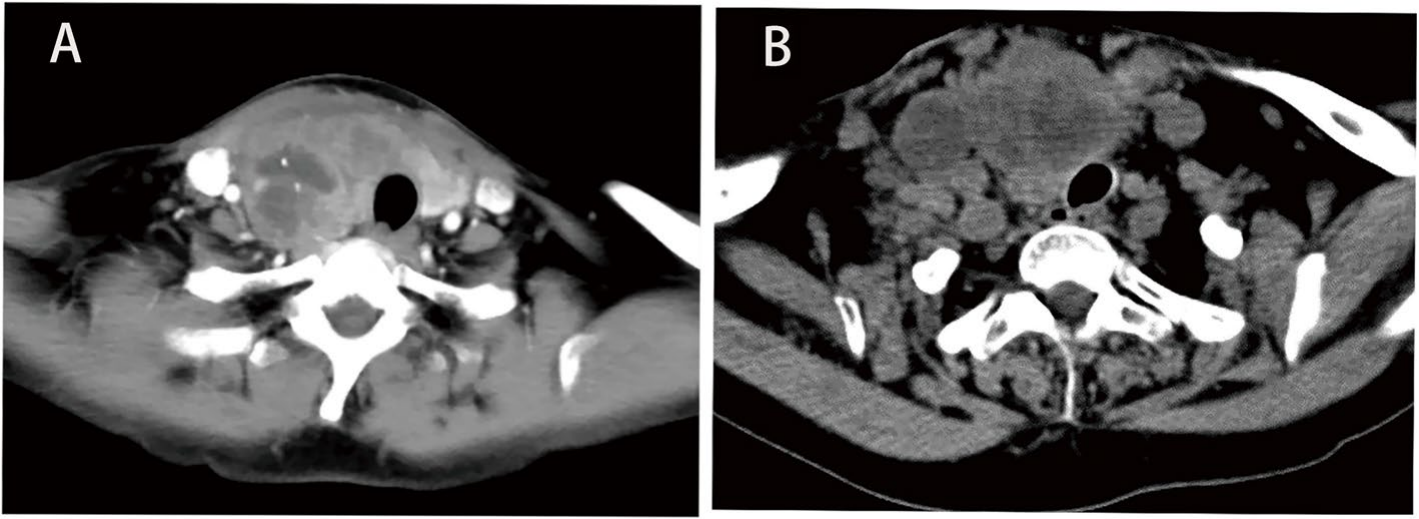
Axial CT image of the neck shows a large right thyroid mass with trachea compression in (**A**) and (**B**), respectively

There were 9 patients who underwent computed tomography (CT) scans in our institution, and all patients presented with a malignant tumor. CT showed that the thyroid gland was enlarged with low-density shadow inside (8/9), irregular shape (4/9), poorly defined boundary (9/9), calcifications (3/9), and necrosis (2/9). The growth of the mass invaded surrounding organs, including the larynx (2/9) and esophagus (2/9). Additionally, 5 patients had tracheal invasion or compression (Fig. 3). Six patients had cervical lymph node metastasis diagnosed by CT scans，4 were confirmed by pathology in the end, and 2 cases without pathology confirmed because they did not undergo neck lymph node dissection.

### Histopathology

In our institution, the diagnosis of all patients was confirmed using histopathology-macroscopic appearance of PSCCT showing a grayish-whitish cancer tissue. In the microscope, thyroid gland tissue showed infiltrative squamous cell carcinoma. Histological evaluation of tumor grade revealed 3 cases of moderate differentiation and 1 case in moderate-low differentiation, yet 7 patients had no determination. Involvement of neck lymph nodes was noted in 85.7% (6/7) of the patients with PSCCT.

### Immunohistochemical features

There were 8 patients in our institution confirmed immunohistochemical staining (Table 2). The Ki67 proliferation index of 5 patients were 20%, 40%, 40%, 60%, and 70%, respectively. In addition, 2 patients tested were positive for thyroid transcription factor 1 (TTF-1) but negative for 3 patients.

**Table 2:** Immunohistochemistry

No.	Immunohistochemistry
**1**	CK5/6(+++), CK(+++), Ki-67 (+40%), TTF-1 (−), Syn(−), CgA(−), CEA(−)
**2**	CK5/6(+), CK19(+), P63(+), TTF-1(−), CT(−), TG(−), Syn(−), CgA(−)
**3**	CK5/6(+++), CK(+++), Ki-67(+20%), CK19(+++), P40(+++), P63(+++), TTF-1(+++), CK14(+), CK18(++), CK7(+++), EMA(++), P53(+++)
**4**	CK5/6(+), CK(+), Ki67 (+70%), CK19(+), P40(+), TTF-1(−), CD5(−), CD117(−), CgA(−), P53(+), P16(−)
**5**	CK5/6(+), CK(+), Ki67 (+60%), CK19(+), P40(+), Gal-3(+), CEA(+), P53(+), AA(+)
**6**	CK5/6(+), CK(+), Ki-67 (+40%), P40(+), P63(+), CK20(−), CK7(−), vimentin(−)
**7**	CK19(+), P40(+), P63(+), TTF-1(+), TG(+), CK7(+)
**8**	CK14(++), CK17(+), TG(+)

*TTF-1* Thyroid transcription factor 1, *CgA* Chromogranin A, *Syn* Synaptophysin, *CEA* Carcinoembryonic antigen, *TG* Thyroglobulin, *EMA* Epithelial membrane antigen, *Gal-3* Galectin-3, *AAT α1*-antitrypsin

### Treatment and prognosis

In our institution, 9 patients underwent surgery, and 2 patients underwent needle biopsy and refused treatment. Additionally, 1 patient underwent radiotherapy, the radiation dose in the residual tumor area was 70 Gy, and the high-risk area was 60 Gy. Two patients underwent chemotherapy. One patient received iodine radiation therapy after surgery due to having papillary thyroid carcinoma synchronously.

**Fig. 4:**
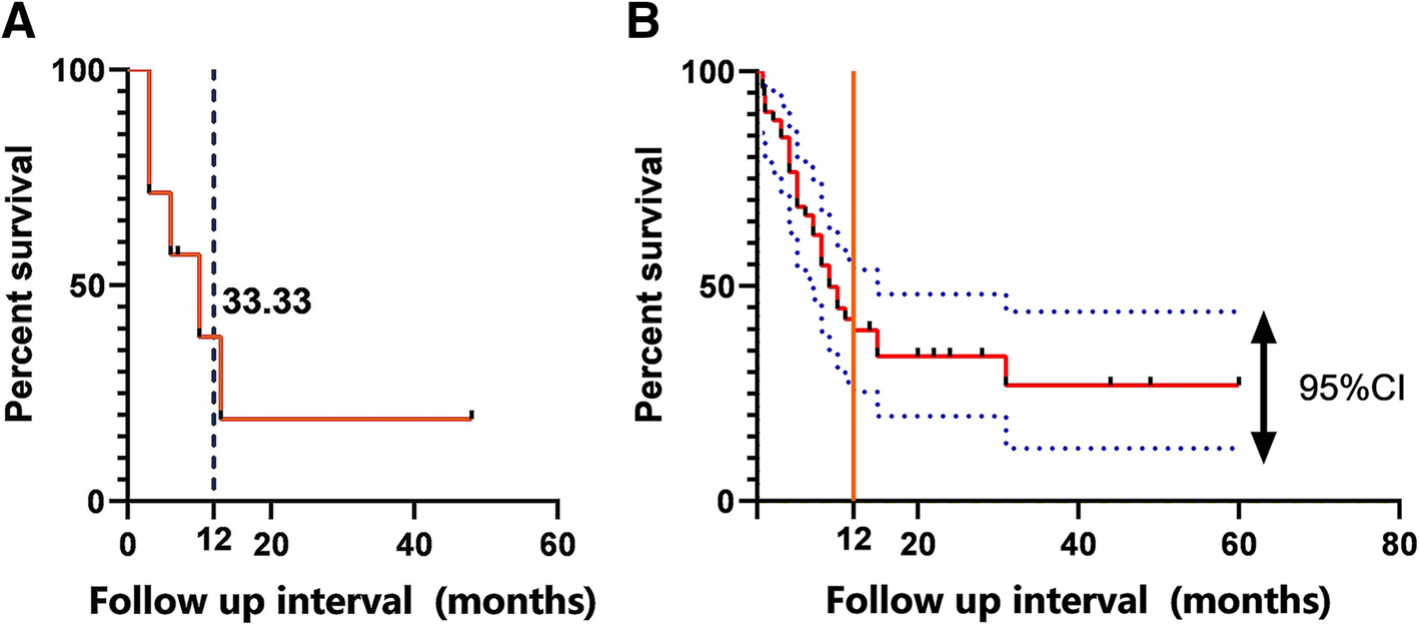
**A** Kaplan–Meier curve for 11 PSCCTs OS. **B** Kaplan–Meier curve for 53 PSCCTs OS. Upper and lower lines indicate 95% confidence interval

**Fig. 5:**
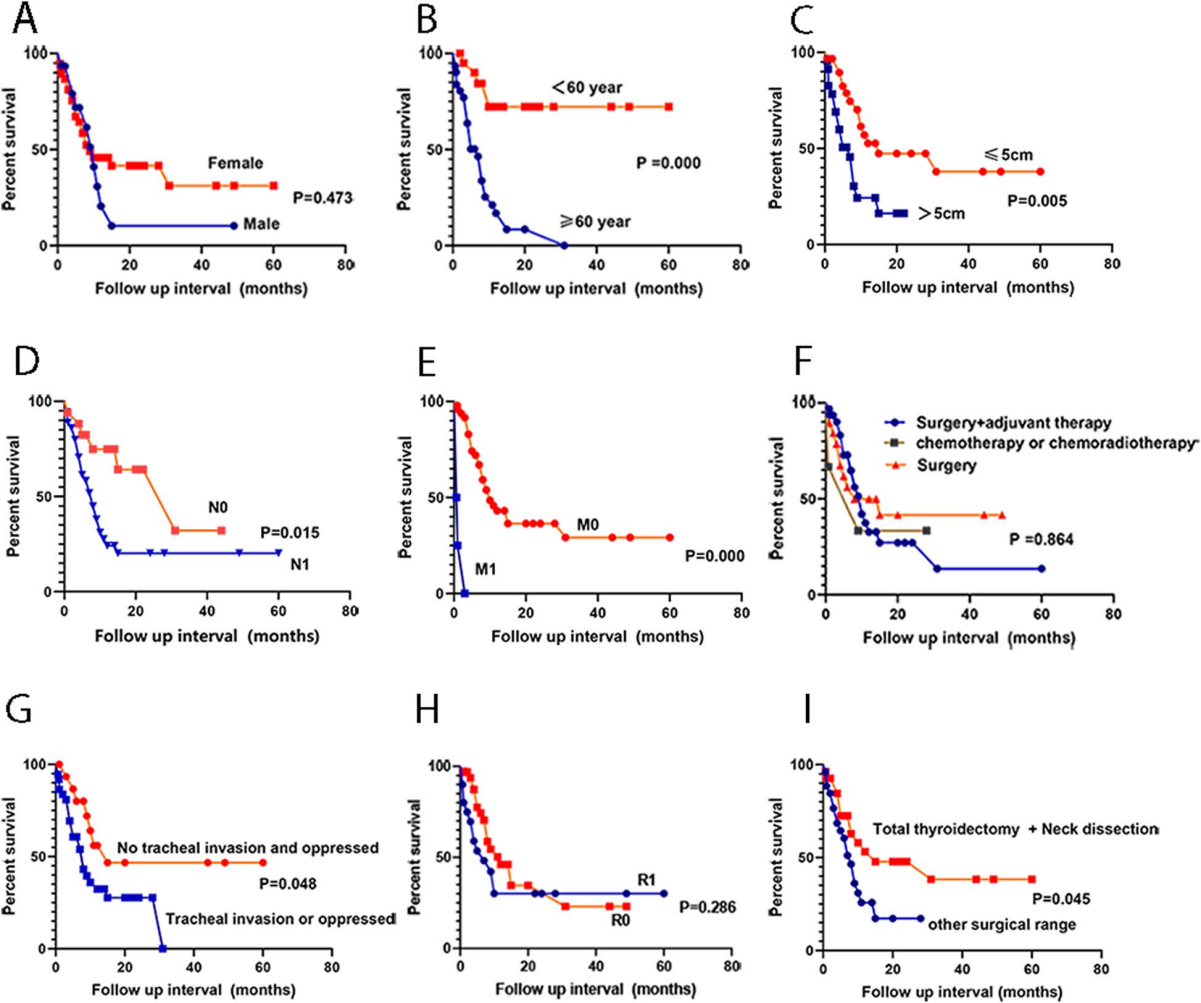
Estimated survival rate by Kaplan–Meier method. **A** Female vs male groups. **B** Age ≥ 60 years vs age < 60y groups. **C** T size > 5 cm vs T size ≤ 5 cm groups. **D** N0 vs N1 groups. **E** No distant metastasis (M0) vs distant metastasis (M1) groups. **F** Surgery alone vs surgery and adjuvant treatment vs chemotherapy (chemoradiotherapy) groups. **G** No tracheal invasion and oppressed vs tracheal invasion or oppressed groups. **H** R0 vs R1 groups. **I** Total thyroidectomy and neck dissection vs other surgical range groups

In conclusion, the estimated survival rate at 1 year by Kaplan–Meier method was 33.3%, and the median OS after diagnosis was 6 months (Fig. 4A). The pooled data of the 53 PSCCTs survival analysis from Kaplan–Meier curves showed that the 1-, 2-, and 5-year overall survival rates were 39.8%, 33.7%, and 26.9% (Fig. 4B). The median OS after diagnosis was 9 months, and average OS was 22 months. As summarized in Table 3, OS showed a statistically significant difference in survival based on age at diagnosis, tumor size, lymph node status, M stage, surgical range, and tracheal status (Fig. 5). In contrast, gender, treatment modality, and resection margin did not show statistically significant differences for predicting OS. Thus, we eliminated these factors in the multivariable analysis, which showed that age and M stage were independent prognostic factors. Relative to age at diagnosis < 60 years, age ≥ 60 years conferred worse OS (*P* = 0.001). Additionally, patients with distant metastasis caused worse OS (*P* = 0.003) (Table 4).

**Table 3:** The results of univariate analysis for prognostic factors among clinical variables (*N* = 53)

Characteristic	No. of patients (%)	Median survival (month)	*p*-value
Gender			
Male	15 (28.3)	10	
Female	38 (71.7)	9	0.473
Age (year)			
<60	22 (41.5)	None	
≥ 60	31 (58.5)	7	0.000
Tumor size (cm)			
≤ 5 cm	30 (56.6)	15	
> 5 cm	23 (43.4)	7	0.005
Lymph node status			
N0	17 (32.1)	31	
N1	36 (67.9)	8	0.015
M stage			
M0	49 (92.5)	10	
M1	4 (7.5)	0.7	0.000
Treatment modality			
Surgery	19 (35.8)	8	
Surgery + adjuvant therapy	31 (58.5)	10	
Chemotherapy or chemoradiotherapy	3 (5.7)	9	0.864
Tracheal invasion or oppressed			
No	16 (30.2)	15	
Yes	37 (69.8)	8	0.048
Resection margin			
R0	33 (62.3)	12	
R1	20 (37.7)	7	0.286
Surgical range			
Total thyroidectomy + neck dissection	26 (49.1)	15	
Others	27 (50.9)	8	0.045

**Table 4:** The results of multivariable analysis for prognostic factors among clinical variables (*N* = 53)

Characteristic	Overall survival HR (95% *CI*)	*p*-value
Age	0.181 (0.065–0.504)	0.001
Tumor size	0.599 (0.221–1.622)	0.314
Lymph node status	0.399 (0.141–1.129)	0.083
M stage	0.111 (0.026–0.486)	0.003
Tracheal invasion or oppressed	0.737 (0.233–2.331)	0.603
Operation	1.318 (0.529–3.283)	0.553

## Discussion

The thyroid gland does not normally contain squamous epithelium. Therefore, the origin of PSCCT remains controversial [5]. Several hypotheses have been proposed, but every theory has been disputed. One of the earliest theories was presented by Goldberg and Harvey in 1956. They pointed out that squamous cell was derived from the embryonic remnants of the thyroglossal duct. In early embryonic life, the thyroid gland migrates downwards, and a duct may persist lined by squamous, columnar, or transitional epithelium. Normally, this duct completely degenerates, but if the duct could not, it might lead to a thyroglossal duct cyst or epidermoid carcinoma [6]. The lowest part of the thyroglossal duct forms the pyramidal lobe of the thyroid gland. If these squamous cells were to give rise to malignancy, then squamous cell carcinoma (SCC) would be expected to arise in the pyramidal lobe of the thyroid gland [6]. However, the preferred location of PSCCT is not the pyramidal lobe of the thyroid gland according to the literature. Secondly, squamous metaplasia is caused by other potential pathological stimuli, such as Hashimoto’s thyroiditis [7]. Thirdly, it is derived from papillary carcinoma, follicular carcinoma, or medullary thyroid carcinoma [8].

Given the rarity of PSCCT, the largest of these studies was from Limberg [2] about 314 cases of PSCCT, treated from 2004 to 2015 and recorded in the American College of Surgeons National Cancer Database. Currently, the understanding of clinical features, molecular manifestations, and treatment is limited. No clear consensus has been reached with regard to optimal therapy for PSCCT [2, 9]. The study found that the prognosis of PSCCT is starkly different from differentiated thyroid malignancies. Instead, PSCCT is often assumed to follow a disease course analogous to anaplastic thyroid carcinoma (ATC) with an equally poor prognosis. Still, compared to ATC, PSCCT patients had smaller tumors and presented with tumors that had fewer associated aggressive features [2]. The median survival of PSCCT was 7.7 months, and the median survival of ATC was 3.8 months [2]. In addition, Kakudo [10] reported undifferentiated carcinoma with a high Ki-67 labeling index (more than 30%). In our institution, the ki-67 index of PSCCT was 20%, 40%, 40%, 60%, and 70%, respectively, which share a similar prognosis equivalent to ATC.

Histopathological and immunohistochemical diagnoses are essential for PSCCT. Kargi [11] reported the TTF-1−, p63+, and CK5/6+ profile with morphologically identifiable squamous differentiation. TTF-1 is a tissue-specific transcription factor that mediates cell determination and differentiation in the lung, thyroid, and brain, expressed in normal thyroid follicular and parafollicular cells [12]. TTF-1 activates the transcription of thyroglobulin, thyroperoxidase, and sodium-iodine transport protein in the thyroid [13–15]. It is considered a marker of differentiation in thyroid and lung carcinomas and a useful immunohistochemical marker in diagnosing these cancers [16, 17]. In the Tan A [18, 19]. report, TTF-1 expressed nearly 100% of papillary thyroid carcinomas, follicular carcinomas, and follicular adenomas. But only approximately 5.7 (2/35) to 18% (5/28) of anaplastic carcinomas are TTF-1 positive [19, 20]. In addition, it is rarely expressed in squamous cell carcinomas [21]. Alfred K [4]. reported TTF-1 positivity in 17% (3/18) of PSCCT cases. Loss of TTF-1 is a nearly uniform feature of PSCCT. But in our study, TTF-1 was reactive in 40% (2/5) of patients. The rate of TTF-1 positivity in PSCCTs was considerably higher than the published positivity rates, which may be related to the fewer cases in this study.

P63 is a transcription factor of the p53 gene family, which regulates the activity of many genes involved in the ectoderm’s growth and development and derived structures and tissues [22]. P63 immunohistochemistry has been widely used as lineage markers for squamous epithelium and squamous cell carcinoma in routine pathology practice. We observed almost 100% of cases (4/4) showed a positive reactions, similar to Alfred K [4].. However, p63 positivity in thyroid tumor is not limited to squamous carcinoma. Tan [18] reported that p63 expression was 41.2% in papillary thyroid carcinoma cases, 28.6% in follicular carcinoma cases, and 66.7% in follicular adenoma cases.

CK5/6 is an intermediate filament of the group of high molecular weight cytokeratins [23] and is also a marker of squamous cell carcinomas [24]. CK5/6 were not detected in normal thyroid parenchyma, lymphocytic thyroiditis, follicular carcinoma, and poorly differentiated carcinoma. But in thyroid papillary carcinoma, expression of CK5/6 was detected in 65.8% (27/41) of the cases [25]. In a study by Handra [26], CK5/6 was expressed in squamoid and basaloid thyroid lymphoepithelial complex. All the primary thyroid squamous cell carcinomas tested in our study showed positivity to CK5/6 (6/6). Based on prior analysis, our experiences were that multiple stains such as p40, p63, TTF-1, and CK5/6 should have been applied to diagnose. At the same time, secondary squamous cell carcinomas originating from adjacent structures by direct invasion or distant metastases from the head, neck, thorax, or upper digestive tract must be excluded. Metastatic SCC can also be poorly differentiated, adding to the difficulty in diagnosis. It has been reported that PAX-8 is positive in PSCCT, whereas squamous cell carcinomas from the cervix, esophagus, and lung were negative for PAX-8 [27].

In our study, the female to male ratio is 1.7–2.5 in PSCCT, with female preponderance consistently with the prior research [1, 4]. Estrogen is a potential growth factor both for benign and malignant thyroid cells which may explain the gender difference in thyroid cancer [28]. Unique among malignancies, age is a key prognostic indicator for well-differentiated thyroid cancer. Similarly, our study confirmed age was a predictor factor for PSCCT. But the association between age and outcome is not entirely clear. Gene rearrangement in older people with PSCCT may dictate tumor aggressiveness and result in a worse prognosis. As such, the presence of the BRAF V600E mutation was significantly associated with increased cancer-related mortality, and this risk increased with age [29]. Interestingly, Ko S. Y [30]. and Brandenburg T [31]. study found PSCCT with a BRAF^V600E^ mutation. Moreover, higher thyroid-stimulating hormone (TSH) in older people increased the likelihood of thyroid cancer [32, 33]. In addition, follicle-stimulating hormone (FSH) and luteinizing hormone (LH) rise with menopause stimulating thyroid cancer growth and invasion by stimulation of the TSH receptor [34].

Currently, surgery is considered an effective treatment option to reduce tumor burden and symptoms of local invasion [1, 3, 35]. Successful surgical resection of PSCCT tumors was associated with improved median OS [2]. In our study, we found that patients in the R0 group, median OS, increased by 5 months compared to the R1 group. However, there is no obvious benefit in the 3-year survival rate because the survival curves crossed after 20 months. We are not the first research team to draw this conclusion. Limberg’s [2] study showed the median OS for patients with complete macroscopic resection was 10.2 months, the median OS for patients performed incomplete macroscopic resection was 3.4 months, and the survival curves intersected after 40 months. We considered that this result might be related to the following reason. Macroscopic resection was in the majority of cases according to the literature recorded. The tumor burden and local compression symptoms were reduced after complete macroscopic resection, so the prognosis of patients in the R0 group is better than the R1 group in the period. However, completing macroscopic resection may have residual cancer cells in the body. But PSCCT is not sensitive to radiotherapy or chemotherapy according to Au JK [3], Cho J. K [35]., and Limberg [2] studies. Therefore, the residual cancer cells gradually grow, resulting in PSCCT recurrence. If patient is not regularly examined, the prognosis becomes worse. At the same time, Limberg [2] found that the median overall survival for patients who achieved microscopic complete tumor resection was 55.5 months, which significantly improved the prognosis (*p* < 0.001). This result confirmed our suppose in a certain. The resection margin may be an independent factor that affects prognosis, but further study is needed, such as adding the subgroup of complete microscopic resection. Unfortunately, such an analysis is beyond the scope of our data. Compared with surgery, whether adjuvant therapy improves the prognosis of PSCCT is still controversial. Au J. K [3]. and Cho J. K [35]. demonstrated no benefit of radiation in conjunction with surgery. In Limberg’s [2] study, neither adjuvant radiation nor chemotherapy showed any survival benefit when at least a complete macroscopic resection was achieved. Chemotherapy and radiation could potentially be considered for PSCCT patients when complete resection is not achieved or unable to be performed, which increased 5 months in median OS. Our analysis concluded that treatment modality is not an independent predictor of OS, but the prognosis of surgery alone is better than surgery combined with adjuvant therapy in terms of 2- and 5-year survival rate, which is consistent with Cho J. K [35]. and Yang [1]. At the same time, there is limited literature about targeted therapy and immunotherapy in PSCCT. Mary Torrez [36] first started on dabrafenib plus trametinib treatment for PSCCT patients with BRAF^V600E^ mutated. Regrettably, the patient continued for 2 weeks with initial clinical benefit but developed increasing fatigue and deconditioning. In contrast, Brandenburg T [31]. recorded a patient with BRAF^V600E^ mutated in PSCCT treated with dabrafenib and trametinib, which had a good quality of life for 12 months. So far, the efficacy of dabrafenib and trametinib treated in PSCCT is not exact, and more studies need to be done.

Considering the PSCCT has a larger tumor size and higher extra thyroid extension, our team supposed tracheal status is related to the prognosis of PSCCT. After all, the trachea is behind the thyroid gland in anatomy. To verify this view, we divided the patients into two groups according to their tracheal status, but we did not obtain the results expectedly.

There are limited data on molecular biology studies of PSCCT. BRAF^V600E^ mutation is present in many human cancers, such as thyroid carcinoma, head and neck squamous cell carcinomas, and gastrointestinal stromal tumors [37]. An activating BRAF mutation is the most common genetic event in thyroid carcinoma, found in roughly 45% of papillary thyroid carcinomas [38]. Similarly, the genomic profile in PSCCT also showed a BRAF mutation [30, 31, 36]. Most studies have demonstrated that BRAF^V600E^ mutation is associated with high-risk histopathologic features, such as an increased frequency of extrathyroidal extension, lymph node metastases, and higher clinical stage [39]. It is associated with the PSCCT clinical features certainly. Chu and colleagues described the mutational characterization from whole exome sequencing of a case of PSCCT, including TMPRSS2, BRCA1, ASPSCR1, and others, but the identified driver mutations common in squamous carcinomas are absent [40]. Further studies are required to investigate mutation mechanisms in PSCCT.

Our study demonstrated patients with PSCCT typically present with rapidly increasing neck mass invading the adjacent structures and with accompanying dysphagia, dyspnea, and hoarseness. Compared with the literature reviewed cases, the patients in our institution seem to have younger onset age, small tumor size, higher malignancy, and worse prognosis. Lymph node positivity was more prevalent in our institution (85.7%), and it was similarly associated with worse median overall survival (6 vs. 9 months). Regrettably, our institution is limited by the case and cannot predict the 2- and 5-year OS rates and analyze survival factors in PSCCT to compare with the literature review results. Further analysis is warranted to determine what drives this difference, such as racial difference. In addition, we were confused about the different prognoses between PTC and PSCCT. The prognosis of PSCCT is much worse than PTC. But there is a lack of molecular evidence to illustrate the difference between PTC and PSCCT. Genomic studies on PSCCT and comparison with ATC or PTC are necessary, so we intend to join forces with other medical institutions, collect more tissue samples for genomic, transcriptomic, proteomic, and metabolomic studies to know molecular changes in PSCCT, and gain insight into the tumorigenicity, disease biology, treatment, and prognosis of PSCCT through multiple levels, which will be very useful to us.

### Limitation

Lack of data is a major limitation in our study. It is difficult to generalize results to a larger population with case studies. In addition, the data collection in our institution is covered 10 years ago, resulting in some patients losing follow-up. The lack of further biomolecular analysis of PSCCT is also a limitation for our studies, such as mutant genes and targeted drugs. Despite these limitations, our study increases the number of PSCCT reported at present, which provides convenience for scholars to study PSCCT in the future. Meanwhile, this manuscript considered the tracheal status and surgical range as variables. To our limited knowledge, published English literature is absent from the relevant study.

## Conclusions

PSCCT is a highly aggressive malignant tumor associated with a poor prognosis. Our retrospective study’s median overall survival was 6–9 months. Age and M stage were related to the prognosis. There is a limited understanding of this tumor’s treatment and molecular biology. More genomic works and relevant perspectives need to be done.

## Supplementary Information

**Additional file 1: Supplemental Table S1.** The results of literature review.

**Additional file 2: Supplemental Table S2.** Search strategy.

## Data Availability

Data are available upon reasonable request to the corresponding author.

## Abbreviations

PSCCT: Primary squamous cell carcinoma of thyroid gland
OS: Overall survival
NED: No evidence of disease
DOD: Died of disease
CT: Computed tomography
TTF-1: Thyroid transcription factor 1
CgA: Chromogranin A
Syn: Synaptophysin
CEA: Carcinoembryonic antigen
TG: Thyroglobulin
EMA: Epithelial membrane antigen
Gal-3: Galectin-3
AAT: α1-Antitrypsin
M0: No distant metastasis
M1: Distant metastasis
SCC: Squamous cell carcinomas
PAX-8: Paired box gene 8
ATC: Anaplastic thyroid carcinoma
PTC: Papillary thyroid carcinoma
P53: Tumor protein 53
P63: Tumor protein 63
CK5/6: Cytokeratin 5/6
TSH: Thyroid-stimulating hormone
LH: Luteinizing hormone
FSH: Follicle-stimulating hormone
BRAF: V-Raf murine sarcoma viral oncogene homolog B1
DTC: Differentiated thyroid carcinoma
TMPRSS2: Transmembrane serine protease 2
BRCA1: Breast cancer susceptibility gene 1
ASPSCR1: Alveolar soft part sarcoma critical region 1
