# Supplementary material for: Primary squamous cell carcinoma of thyroid gland: 11 case reports and a population-based study
Source: World J Surg Oncol. 2022 Nov 3;20:352. doi: 10.1186/s12957-022-02814-9 (PMC9632099; doi:10.1186/s12957-022-02814-9)
Supplement: Supplementary file 1 — Additional file 1: Supplemental Table S1. The results of literature review. [file 12957_2022_2814_MOESM1_ESM.doc]

|  | **Author (Year)**  Supplemental Table S1. The results of literature review | **Tittle** | **Patient numbers** | **Age/gender** | **Outcome** |
| --- | --- | --- | --- | --- | --- |
| 1 | Saito K et al. (1981) | Primary squamous cell carcinoma of the thyroid associated with marked leukocytosis and hypercalcemia | 1 | 71/F | 1 |
| 2 | Segal K et al. (1984) | Pure squamous cell carcinoma and mixed adenosquamous cell carcinoma of the thyroid gland | 2 | 47/F  73/F | 0  1 |
| 3 | Kapoor VK et al. (1985) | Primary squamous cell carcinoma of the thyroid gland a case report | 1 | 45/F | 0 |
| 4 | Misonou J et al. (1988) | Pure squamous cell carcinoma of the thyroid gland report of an autopsy case and review of the literature | 1 | 61/F | 1 |
| 5 | Theander C et al. (1993) | Primary squamous carcinoma of the thyroid a case report | 1 | 72/F | 1 |
| 6 | Zhenmu Z et.al (1996) | Primary thyroid squamous cell carcinoma three cases report | 1 | 64/F | 1 |
| 7 | Bing Z（1999） | Primary squamous carcinoma of the thyroid a case report | 1 | 68/M | 1 |
| 8 | Sahoo M et al. (2002) | Primary squamous cell carcinoma of the thyroid gland new evidence in support of follicular epithelial cell origin | 2 | 42/F  55/F | 1  1 |
| 9 | Qilong S et al. (2005) | Primary thyroid squamous cell carcinoma a case of report | 1 | 54/F | 0 |
| 10 | Chintamani KP et al. (2007) | Is an aggressive approach justified in the management of an aggressive cancer the squamous cell carcinoma of thyroid | 1 | 50/F | 1 |
| 11 | Eom TI et al.  (2008) | Coexistence of primary squamous cell carcinoma of thyroid with classic papillary thyroid carcinoma | 1 | 43/F | 0 |
| 12 | Makay O et al. (2008) | Primary squamous cell carcinoma of the thyroid: report of three cases | 1 | 71/M | 1 |
| 13 | Joo YH et al. (2008) | A Case of Synchronous Squamous Cell and Papillary Carcinoma of the Thyroid Gland | 1 | 69/M | 1 |
| 14 | Rausch Th et al. (2009) | Thyroid carcinoma with papillary and squamous features report of a case with histogenetic considerations | 1 | 82/F | 1 |
| 15 | Yucel H et al. (2010) | Primary squamous cell carcinoma of the thyroid years after radioactive iodine treatment | 1 | 88/M | 0 |
| **The results of literature review** | **Author (Year)** | **Tittle** | **Patient numbers** | **Age/gender** | **Outcome** |
| 16 | Rodríguez J et al. (2010) | Carcinoma de Células Escamosas de Tiroides: A Propósito de un Caso (Primary squamous cell thyroid carcinoma) | 1 | 67/M | 1 |
| 17 | Kim HS et al.  (2011) | A Case of Mixed Papillary Thyroid Tumor and Squamous Cell Carcinoma | 1 | 30/F | 0 |
| 18 | Tao J et al.  (2011) | Primary thyroid squamous cell carcinoma a case report and literature review | 1 | 46/M | 0 |
| 19 | Ko YS et al.  (2012) | Primary pure squamous cell carcinoma of the thyroid report and histogenic consideration of a case involving a BRAF mutation | 1 | 87/M | 1 |
| 20 | KH Chen et al.  (2012) | Primary squamous cell carcinoma of the thyroid with cardiac metastases and right ventricle outflow tract obstruction | 1 | 67/F | 1 |
| 21 | De Vos et al. (2012) | Combined therapy for thyroid squamous cell carcinoma | 1 | 51/F | 0 |
| 22 | Choi JS et al.  (2012) | Analysis of Primary Squamous Cell Carcinoma of Thyroid | 6 | 24/F  84/F  41/M  55/F  76/F  33/F | 0  1  0  1  1  0 |
| 23 | Evans G (2012) | Dedifferentiation of papillary thyroid carcinoma into squamous cell carcinoma. A case of coexistence within an excised neck lesion | 1 | 80/M | 1 |
| 24 | Shrestha M et al.（2013） | Primary squamous cell carcinoma of the thyroid gland: a case report and review | 1 | 75/F | 1 |
| 25 | Lee JI et al.  (2013) | Papillary Thyroid Carcinoma Recurring as Squamous Cell Carcinoma 10 years after Total Thyroidectomy: Lessons from Rapidly Progressive Papillary Thyroid Carcinoma | 1 | 86/F | 1 |
| 26 | Ko JS et al.  (2013) | Two Cases of Primary Papillary Thyroid Cancer with a Squamous Cell Carcinoma Component | 2 | 82/F  30/F | 1  0 |
|  | **Author (Year)** | **Tittle** | **Patient numbers** | **Age/gender** | **Outcome** |
| 27 | Manrique FK et al. (2014) | Papillary thyroid carcinoma associated to squamous cell carcinoma | 1 | 62/F | 1 |
| 28 | Sapalidis K et al. (2014) | Primary squamous cell carcinoma of the thyroid gland | 1 | 65/F | 1 |
| 29 | Da HS et al. (2014) | Combined Squamous Cell Carcinoma and Follicular Carcinoma of the Thyroid | 1 | 69/M | 1 |
| 30 | Feng Z et al. (2014) | Primary squamous carcinoma of the thyroid a case report | 1 | 61/F | 0 |
| 31 | JianGuo Z et al. (2014) | Primary thyroid squamous cell carcinoma with distant metastasis: a case report | 1 | 65/F | 1 |
| 32 | Yan K et al.  (2014) | Diagnosis and treatment of primary thyroid squamous cell carcinoma with a typical case report and literature review | 1 | 66/F | 1 |
| 33 | Yuqi H et al.  (2014) | Primary thyroid squamous cell carcinoma: a case report and literature review | 1 | 58/M | 0 |
| 34 | Yanle W et al.  (2014) | Papillary thyroid carcinoma with squamous cell carcinoma and Warthin tumor formation a case report | 1 | 63/F | 0 |
| 35 | Jinling H et al.  (2014) | Primary thyroid squamous cell carcinoma a case report | 1 | 72/M | 1 |
| 36 | Dong S et al.  (2016) | Mixed primary squamous cell carcinoma, follicular carcinoma, and micropapillary carcinoma of the thyroid gland A case report | 1 | 62/F | 0 |
| 37 | YuXian W et al. (2016) | Primary thyroid papillary carcinoma combined with squamous cell carcinoma report of one case and a review of literature | 1 | 69/M | 1 |
| 38 | Wygoda A et al. (2017) | Primary squamous-cell thyroid carcinoma a successful treatment with five-year follow-up | 1 | 42/F | 0 |
| 39 | Kallel S et al. (2018) | Primary squamous cell carcinoma of the thyroid associated with papillary thyroid carcinoma and Hashimoto's thyroiditis | 1 | 54/F | 0 |
|  | **Author (Year)** | **Tittle** | **Patient numbers** | **Age/gender** | **Outcome** |
| 40 | Mohd I et al.  (2018) | Primary Squamous Cell Carcinoma of the Thyroid Gland | 1 | 74/F | 1 |
| 41 | De CA et al. (2019) | Total thyroidectomy associated to chemotherapy in primary squamous cell carcinoma of the thyroid | 1 | 58/M | 1 |
| 42 | Akbar S et al. (2021) | Primary thyroid squamous cell carcinoma presenting as a left-sided neck lump | 1 | 83/F | 0 |
| 43 | Chu MMH et al. (2021) | Primary squamous cell carcinoma of the thyroid gland successfully treated with surgical resection and adjuvant chemoradiotherapy | 1 | 57/F | 0 |
| 44 | Soror NN et al.（2021） | Primary Squamous Cell Carcinoma of the Thyroid: A Case Report and Literature Review About a Rare Entity | 1 | 53/M | 0 |
| 45 | Sho Iwaki et al. (2021) | Long-term efficacy of weekly paclitaxel therapy in unresectable primary squamous cell carcinoma of the thyroid | 1 | 59/F | 0 |

(Outcome: 1 represent death; 0 represent alive)
