# Supplementary material for: Primary squamous cell carcinoma of thyroid gland: 11 case reports and a population-based study
Source: World J Surg Oncol. 2022 Nov 3;20:352. doi: 10.1186/s12957-022-02814-9 (PMC9632099; doi:10.1186/s12957-022-02814-9)
Supplement: Supplementary file 2 — Additional file 2: Supplemental Table S2. Search strategy. [file 12957_2022_2814_MOESM2_ESM.doc]

| Search | Query（ before Dec 22, 2021） | Items  found |
| --- | --- | --- |
| PubMed | **Search: ((((Thyroid Carcinomas) OR (Thyroid Neoplasm)) OR (Thyroid Cancers)) AND (Squamous Cell Carcinoma)) AND (Primary)**  ("thyroid neoplasms"[MeSH Terms] OR ("thyroid"[All Fields] AND "neoplasms"[All Fields]) OR "thyroid neoplasms"[All Fields] OR ("thyroid"[All Fields] AND "carcinomas"[All Fields]) OR "thyroid carcinomas"[All Fields] OR ("thyroid neoplasms"[MeSH Terms] OR ("thyroid"[All Fields] AND "neoplasms"[All Fields]) OR "thyroid neoplasms"[All Fields] OR ("thyroid"[All Fields] AND "neoplasm"[All Fields]) OR "thyroid neoplasm"[All Fields]) OR ("thyroid neoplasms"[MeSH Terms] OR ("thyroid"[All Fields] AND "neoplasms"[All Fields]) OR "thyroid neoplasms"[All Fields] OR ("thyroid"[All Fields] AND "cancers"[All Fields]) OR "thyroid cancers"[All Fields])) AND ("carcinoma, squamous cell"[MeSH Terms] OR ("carcinoma"[All Fields] AND "squamous"[All Fields] AND "cell"[All Fields]) OR "squamous cell carcinoma"[All Fields] OR ("squamous"[All Fields] AND "cell"[All Fields] AND "carcinoma"[All Fields])) AND ("primaries"[All Fields] OR "primary"[All Fields]) | 703 |
| Web of science | ((((Thyroid Carcinomas) OR (Thyroid Neoplasm)) OR (Thyroid Cancers)) AND (Squamous Cell Carcinoma)) AND (Primary)(Topic) | 1105 |
| ProQuest | su(((((Thyroid Carcinomas) OR (Thyroid Neoplasm)) OR (Thyroid Cancers)) AND (Squamous Cell Carcinoma)) ) OR ab(((((Thyroid Carcinomas) OR (Thyroid Neoplasm)) OR (Thyroid Cancers)) AND (Squamous Cell Carcinoma))) | 630 |
| Embase | ('Thyroid Carcinomas' OR 'Thyroid Neoplasm' /exp OR 'Thyroid Cancers') AND 'Squamous Cell Carcinoma'/exp AND Primary | 754 |

Supplementary file 2 Table S2. Search strategy

| Search | Query（ before Dec 22, 2021） | Items  found |
| --- | --- | --- |
| CNKI | 关键词：(甲状腺 * (鳞状细胞癌 + 鳞癌) * 原发性)（精确）） OR 摘要：(甲状腺 * (鳞状细胞癌 + 鳞癌) * 原发性)（精确）） OR 篇名：(甲状腺 * (鳞状细胞癌 + 鳞癌) * 原发性)（精确））  Translation: Keywords: (thyroid* (squamous cell carcinoma + squamous carcinoma) * primary) (exact)) OR abstract: (thyroid * (squamous cell carcinoma + squamous carcinoma) * primary) (exact)) OR Tittle: (thyroid* (squamous cell carcinoma + squamous carcinoma) * primary) (exact)) | 214 |
| VIP | 关键词：((甲状腺 and (鳞状细胞癌 or 鳞癌) and 原发性)） or 摘要： ((甲状腺 and (鳞状细胞癌 or 鳞癌) and 原发性)） or 篇名： ((甲状腺 and (鳞状细胞癌 or 鳞癌) and 原发性)）  Translation: Keywords: (thyroid AND (squamous cell carcinoma OR squamous carcinoma) AND primary)) OR abstract: (thyroid AND (squamous cell carcinoma OR squamous carcinoma) AND primary)) OR Tittle: (thyroid AND (squamous cell carcinoma OR squamous carcinoma) AND primary)) | 141 |
| Wanfang | 关键词：((甲状腺 and (鳞状细胞癌 or 鳞癌) and 原发性)） or 摘要： ((甲状腺 and (鳞状细胞癌 or 鳞癌) and 原发性)） or 篇名： ((甲状腺 and (鳞状细胞癌 or 鳞癌) and 原发性)）  Translation: Keywords: (thyroid AND (squamous cell carcinoma OR squamous carcinoma) AND primary)) OR abstract: (thyroid AND (squamous cell carcinoma OR squamous carcinoma) AND primary)) OR Tittle: (thyroid AND (squamous cell carcinoma OR squamous carcinoma) AND primary)) | 198 |
